# Feeding without teeth: the material properties of rhamphothecae from two species of durophagous sea turtles

**DOI:** 10.1098/rsos.221424

**Published:** 2023-04-19

**Authors:** Danielle N. Ingle, Eliza Perez, Marianne E. Porter, Christopher D. Marshall

**Affiliations:** ^1^ Department of Marine Biology, Texas A&M University at Galveston, Galveston, TX 77554, USA; ^2^ Gulf Center for Sea Turtle Research, Texas A&M University, College Station, TX 77843, USA; ^3^ Department of Ecology and Conservation Biology, Texas A&M University, College Station, TX 77843, USA; ^4^ Department of Biological Sciences, Florida Atlantic University, Boca Raton, FL 33431, USA

**Keywords:** cornified keratin, bone mineral density, water absorption, Young's modulus, yield strength, toughness

## Abstract

The feeding apparatus of sea turtles comprises cornified keratinous rhamphothecae overlaying a bony rostrum. Although keratin is less stiff than the enamel of toothed animals, certain species of sea turtles are capable of withstanding large forces when feeding on hard prey. We aimed to quantify the mineral density, water content and compressive mechanical properties of rhamphothecae from two durophagous species: loggerhead (*Caretta caretta*) and Kemp's ridley (*Lepidochelys kempii*) sea turtles. Since loggerheads theoretically produce the greater bite forces of these two species, we predicted that keratin from their rhamphothecae would have a greater mineral density and be stiffer, stronger and tougher compared with Kemp's ridley sea turtles. We found that total water weight of hydrated specimens (20%) was consistent between species. Rhamphotheca mineral density ranged between 0 and 0.069 g cm^−3^; loggerheads had significantly greater mineral density compared with Kemp's ridleys, for which several specimens had no mineral detected. Despite the greater mineral density in loggerheads, we found no significant difference in Young's modulus, yield strength or toughness between these species. In addition to mineral density, our findings suggest that other material components, such as sulfur, may be influencing the material properties of keratin from sea turtle rhamphothecae.

## 1. Introduction

Feeding is essential to ensure an animal's survival, reproductive success, and fitness. Access or constraints to certain prey items is largely determined by the morphology and biomechanics of the feeding apparatus [1,2]. Toothed feeding apparatuses were nearly ubiquitous in tetrapods prior to the Late Cretaceous; however, edentulism, and the concomitant appearance of cornified keratinous beak constituents (rhamphothecae), arose independently in several lineages and is now prominent in extant birds and turtles [3–5]. Despite the relatively extreme stiffness of tooth enamel (e.g. 84 GPa, human) [6] compared with the keratin of rhamphothecae (e.g. 1.2 GPa, hornbill) [7], several beaked taxa are capable of consuming hard prey [8–11]. While previous studies have investigated the material properties of avian rhamphothecae [7,12,13], few have focused on the implications of durophagy for keratin material behaviour and composition. Here, we examine the material properties of rhamphothecae from sea turtles, many of which prey on hard-shelled molluscs and crabs, as an ideal model system for investigating the capabilities and constraints of durophagous feeding [10,14–16].

Hard keratinous materials are ubiquitous among tetrapods and include either α-helix or β-sheet intermediate filaments (IFs) embedded in an amorphous keratin matrix. For example, mammalian hair, horns and whale baleen are made of α-helix IFs, while avian and reptilian feathers, claws and beaks are composed of β-sheet IFs [17,18]. Mineral deposition throughout the hard keratinous matrix has been hypothesized to influence material behaviour; Hieronymus *et al*. [19] found that white rhinoceros (*Ceratotherium simum*) horns incorporated calcium phosphate salts, which may increase material hardness and compressive stiffness as well as maintain the conical horn shape. Calcified keratin may also contribute to appendage structural integrity in marine environments; since whale baleen is not able to fully dry throughout the duration of these animals' lives, their calcified baleen may provide sufficient stiffness for filter feeding [20]. While these data highlight how calcified keratinous structures aid in foraging behaviours, there is minimal information regarding mineral content in the rhamphothecae of beaked tetrapods, especially durophagous species [21].

Environmental factors play a key role in keratin material behaviour; hydration levels significantly influence the mechanical properties of hard keratin [18,22]. Zhang *et al*. [23] found that when bovid horn keratin was compressed in an ambient dry condition, it was stiffer, stronger and tougher than fully hydrated samples by factors of over four, six and two, respectively. Similarly, whale baleen had five times greater flexural strength and three times greater flexural stiffness in the ambient dry condition compared with the fully hydrated condition [24]. The influence of hydration levels on keratin material behaviour has implications for aquatic beaked tetrapods, who forage while immersed in water.

Our work focused on two sea turtle species with durophagous diets: loggerheads (*Caretta caretta*) and Kemp's ridleys (*Lepidochelys kempii*). Mature loggerheads forage on crabs, molluscs, barnacles, gelatinous zooplankton, squid and fish in coastal neritic habitats [14,25]. Loggerheads, who have broad heads (hence the name, ‘loggerhead’) and large cross-sectional areas of jaw-closing muscles (i.e. *m. adductor mandibulae*), are arguably capable of crushing through harder prey items than Kemp's ridleys based on head morphometrics and muscle size alone, even after correcting for animal size. Marshall *et al*. [10] found that the largest adult loggerheads had measured bite forces of up to 1766 N, enough to crush through queen conch (*Strombus gigas*) shells [10,26]. Kemp's ridleys also prey on molluscs (i.e. snails) and fish, but mostly feed on crabs: 93% of the dry mass of stomach contents found in Kemp's ridleys from Texas waters consisted of crab [14,16]. Like many sea turtle species, loggerheads and Kemp's ridleys undergo drastic shifts in habitats and diet throughout development [27]. Hatchlings travel along oceanic gyres with an abundance of *Sargassum* algae, which provides a community of organisms as a food resource and cover for predator evasion [28–30]. Their subsequent years are spent in an oceanic phase and omnivorous juveniles exploit a variety of small fishes, invertebrates and gelatinous zooplankton [15,31]. When loggerheads and Kemp's ridleys reach a size of 40–60 cm standard carapace length (SCL) and 20–25 cm SCL, respectively, they recruit to coastal neritic habitats and begin to feed on hard prey [14,27].

Here, we measure and calculate the material properties of rhamphothecae from loggerhead and Kemp's ridley sea turtles in specimens large enough to employ durophagous feeding. We quantified mineral density and water content (in various states of hydration) of intact rhamphothecae, and assessed the mechanical properties of rhamphotheca keratin to understand material rigidity (stiffness; i.e. Young's modulus), stress at permanent deformation (yield strength) and the ability to absorb energy (toughness). In this paper, we collectively refer to the mineral density, water content and mechanical property data as material properties. We then compared water content and mechanical property data from this study with previous literature on cornified keratin to understand these relationships in a comparative context. Since loggerhead sea turtles produce larger measured bite forces, we hypothesized their rhamphothecae would have higher mineral density and greater mechanical properties (i.e. Young's modulus, yield strength and toughness) compared with Kemp's ridley sea turtles. Based on other studies, we hypothesized that increased mineral density would result in increased mechanical properties in both species. Due to the amount of time that both of these species spend in water, we expected their water absorption capacities to be similar.

## 2. Experiments and methods

### 2.1. Materials preparation

**Figure 1.**
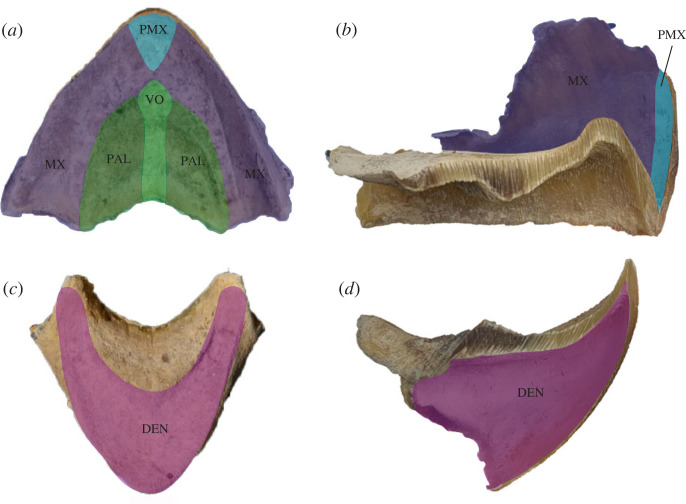
Loggerhead sea turtle upper and lower rhamphothecae in the dorsal/ventral (*a*,*c*) and sagittal views ((*b*,*d*); medially bisected). Coloration denotes underlying bones of the upper and lower jaws. PMX = premaxilla; MX = maxilla; VO = vomer; PAL = palatine; DEN = dentary [34,35].

All samples were collected from necropsied sea turtles that were either found deceased during stranding responses or died in rehabilitative care due to unrecoverable injury or illness. Necropsies were conducted by the Gulf Center for Sea Turtle Research (Texas A&M University, College Station, Texas, USA) under USFWS Permit TE776123-1, and by the Sea Turtle Science and Recovery Division of the Padre Island National Seashore (National Park Service; Corpus Christi, Texas, USA) under USFWS Permit TE840727-3. We collected whole heads from loggerheads and Kemp's ridleys that had greater than or equal to 60 cm SCL and greater than or equal to 25 cm SCL, respectively. Specifically, this study included seven loggerheads (SCLs = 59.5–84.4 cm; *x̄* = 70.5 ± 3.84 cm) and eight Kemp's ridleys (SCLs = 28.5–6.25; *x̄* = 45.8 ± 5.09 cm) that included animal IDs and known SCLs (electronic supplementary material, table S1). Two unidentified loggerheads, whose rhamphotheca sizes indicated that they had SCLs of at least 60 cm, where also included in this study. All animals were large enough to be in the size range of when these species shift to neritic habitats and employ durophagy as a feeding mode [14–16]. Specimen heads were removed at the atlanto-occipital joint using standard dissection equipment and then macerated until soft tissue separated from the skull [32,33]. Maceration is standard in keratinous specimen preparation and was the preferred method of separating beaks since it had the least chance of changing the shape of the rhamphothecae or damaging the keratin. Once maceration was complete, rhamphothecae were removed from the bony beak without damaging either the bone or keratinous tissue (figure 1). Rhamphothecae were then cleaned, air dried and stored at room temperature (22°C) [23]. Prior to data collection, all rhamphothecae were inspected for pathologies, deformities or compromised tissue, none of which were found.

### 2.2. Micro computed tomography scanning

Fifteen sets of rhamphothecae (upper and lower rhamphotheca; loggerhead *n* = 7; Kemp's ridley *n* = 8) were transported to Florida Atlantic University's A.D. Henderson University School (Boca Raton, FL, USA) for data collection. For scanning preparation, rhamphothecae were either wrapped in gauze, packed in custom three-dimensional-printed plastic canisters and secured with plastic wrap to a Bruker Skyscan scanning base (Billerica, MA, USA), or wrapped in gauze and plastic wrapped directly to the scanning base, as necessary. Every scan included two densitometry phantoms, which were packed in with the gauze surrounding the rhamphothecae. These densitometry phantoms had known densities of calcium hydroxyapatite (CaHA; 0.25 g cm^−3^ and 0.75 g cm^−3^) [36]. Micro computed tomography (microCT) scans were conducted using a Bruker Skyscan 1173 (Billerica, MA, USA) at the following ranges of resolutions, voltages and amperages, respectively: 50–68 µm, 36–45 kV and 98–200 µA. Scan resolutions were partially determined by specimen sizes [37].

**Figure 2.**
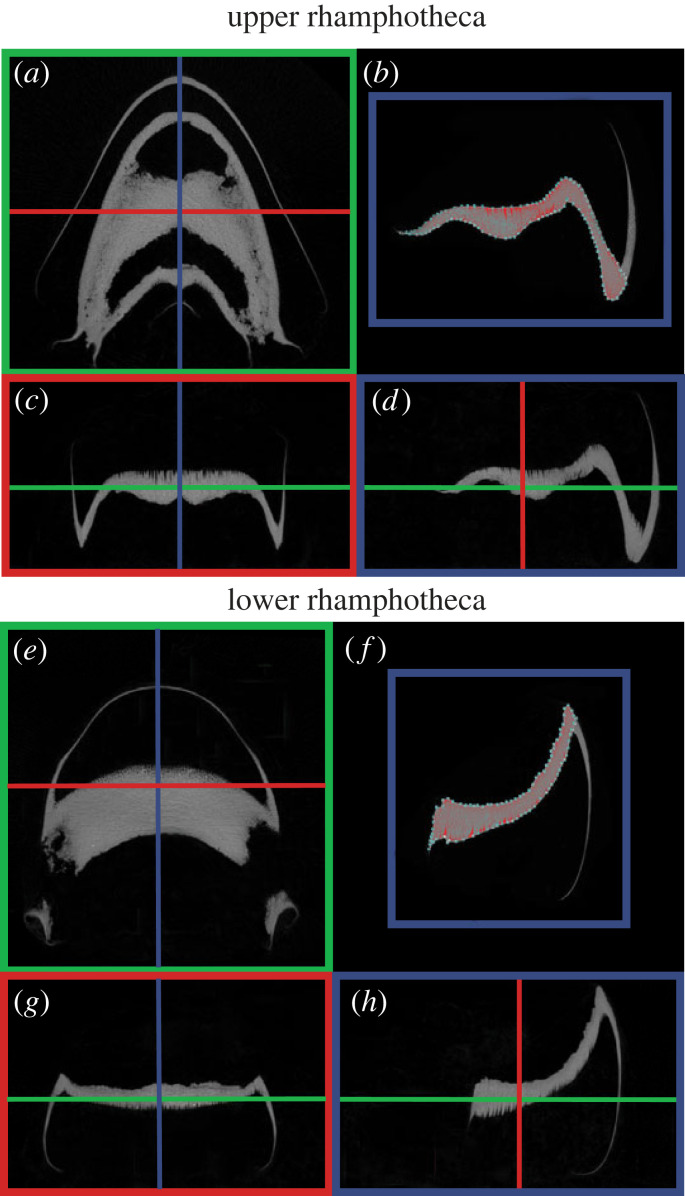
Rhamphotheca microCT scan segmentation (ROI selection for calculating BMD). Bruker Dataviewer software was used to view transverse ((*a*,*e*); green), coronal ((*c*,*g*); red) and sagittal ((*d*,*h*); blue) CT data slices of both upper and lower rhamphothecae. ROIs were selected and BMD calculated from sagittally oriented data slices ((*b*,*f*); blue) using Bruker CTAn software.

CT data were reconstructed using Bruker NRecon software, and each rhamphotheca was segmented using Bruker Dataviewer software and oriented so that slice data bisected rhamphothecae in the sagittal view. Using Bruker CTAn software, regions of interest (ROI) were drawn around the components of the rhamphothecae that cover the premaxilla, maxillae, vomer and palatine bones in the upper rhamphotheca, and the dentary bone in the lower rhamphotheca (figure 1) [34,35]. The proximal boundary of the tips of the upper and lower rhamphothecae (which cover the tips of the premaxillary and dentary bone, respectively) was selected based on the continuation of the ROI line drawn along the deep layer of each rhamphotheca (figure 2). Starting from the left lateral-most point of each rhamphotheca, ROIs were drawn every several data slices (i.e. every 20 for loggerheads and every 15 for Kemp's ridleys) and then interpolated throughout the remaining slices of the scan. The Kemp's ridleys specimens were assigned a relatively higher sampling rate to ensure capture of shape changes in their relatively smaller rhamphothecae. In addition, this frequency of ROI selection allowed us to inspect slice data for any physical damage or abnormalities in the specimens, of which there were none. Bone mineral density (BMD), or the volumetric density of CaHA in a biological tissue, was calculated to quantify any mineralization present throughout the keratinous matrix. BMD for each dataset was calibrated based on the attenuation coefficients of the 0.25 and 0.75 g cm^−3^ CaHA densitometry phantoms.

### 2.3. Water content characterization

Water absorption tests were performed on a subset of six rhamphothecae (Kemp's ridley *n* = 3; loggerhead *n* = 3; electronic supplementary material, table S2). The specimens, which had been air dried for more than 7 days, were weighed to the nearest 0.1 g before being soaked for 5 days in filtered seawater. Every 24 h, rhamphothecae were removed from the water, patted dry with a cloth, weighed and re-immersed in fresh filtered seawater [23]. Rhamphothecae were removed from water after 5 days when there was no increase in specimen weight between the 4th and 5th day of soaking. After the rehydration component of the experiment, specimens were air dried for several days. Once they had returned to their weight before rehydration (± 0.5 g), rhamphothecae were dried in an Isotemp Oven 280A (Lynwood, WA, USA) for 24 h at 110°C then weighed. This drying protocol removed all hydration from cornified keratin [38]. No specimens that were involved in the rehydration and dehydration experiments were included in the separate mechanical tests (see below). Additionally, two sets of loggerhead rhamphothecae that were used for water content characterization were not microCT scanned.

### 2.4. Compression tests

**Figure 3.**
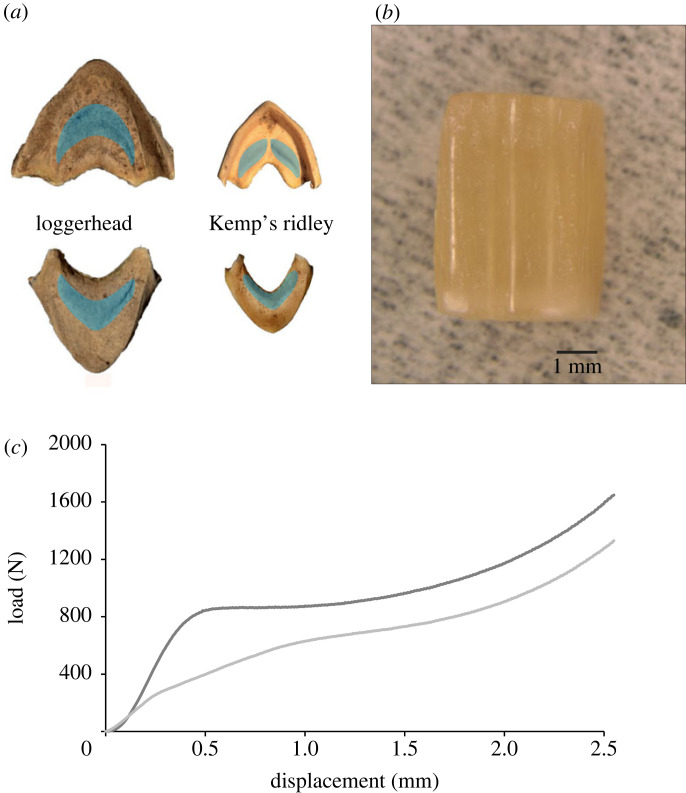
Sample selection and compression tests of keratin. (*a*) Rhamphothecae regions from which keratin was sampled. (*b*) Sagittal view of a keratinous sample. (*c*) Example comparative load-displacement curves for samples taken from lower rhamphothecae of loggerhead and Kemp's ridley sea turtles.

Rhampthothecae selected for compression testing were mid-sagittally bisected using a large Dremel cutting tool (Mt. Prospect, IL, USA; loggerhead *n* = 6; Kemp's ridley *n* = 4). Initially, we had attempted to bisect specimens with a bandsaw, but this cutting mode was not adequate since it lacked accuracy and control relative to the Dremel, and the bandsaw appeared to compromise the keratin. Samples were cut from the thickest portion of each bisected half from both the upper and lower rhamphothecae for a total of four samples for every animal (figure 3*a*). Small Dremel cutting and sanding accessories were then used to ensure each sample had a 5 × 3 × 3 mm size: a minimum of 1 mm was shaved off the superficial and deep layers and excess keratin was removed from the four orthogonal sides. Attention was given to ensure that refinement of the final sample size using these tools was minimal, fast and did not generate heat. Although the removal of the rough, uneven superficial layer might affect mechanical properties, this portion was removed to optimize compression testing accuracy [38]. Samples were cut and sanded in the ambient dry condition [23,38]. For testing preparation, finished samples were immersed in filtered seawater for 5 days to fully rehydrate (figure 3*b*). Filtered seawater was changed daily over the course of the rehydration period [23]. Previous studies found that compression tests on dry keratin produced greater mechanical properties than samples that were fully rehydrated, and testing while hydrated is biologically relevant in these aquatic species [23,38].

After rehydration, samples were blotted dry and placed at the centre of a stationary platen fixed to the base of an MTS Insight 5 material testing system (Eden Prairie, MN, USA). To reduce sample slippage during unconfined uniaxial compression tests, 300 grit sand paper was attached to the base platen's surface. Using a 2.5 kN load cell, the samples were compressed in the dorsoventral orientation (in the context of rhamphothecae) until either the load limit or displacement limit was reached on the testing system. The strain rate of all tests was 2 mm min^−1^, which was informed by previous compressive tests on cornified keratin [39]. Load-displacement data were collected at a frequency of 10 Hz and then converted to stress–strain curves (figure 3*c*). Young's modulus was calculated in TestSuite software (MTS, Eden Prairie, MN, USA) as the steepest portion of the linear portion of the stress–strain curve. Yield strength was calculated at the 0.2% offset, and toughness was measured as the area under the stress–strain curve through 50% strain. The toughness calculation is adapted and modified from Zhang *et al*. [23], who instead quantified the area under the curve through 70% strain. However, our samples rarely exceeded a deformation of 50–60% strain before reaching the load cell or displacement limits; therefore, we standardized our measurements at 50%.

### 2.5. Statistical analysis

All data were tested for normality using the Shapiro–Wilk test. For BMD data, which did not fit a normal distribution, a non-parametric Mann–Whitney test was used to determine differences between loggerhead and Kemp's ridley rhamphothecae. We used a MANOVA to evaluate any interspecific variations in normally distributed water content data (fully dehydrated – ambient dry, ambient dry – fully hydrated, and total water content). For normally distributed compression test data (i.e. Young's modulus, yield strength and toughness), we used a MANOVA to determine if interspecific differences were present. Simple regressions were run between SCL (cm) and all aforementioned data to test for correlation to, and a potential effect of, animal size. The significance criterion for statistical analyses, which were conducted in JMP Pro 16 (Cary, NC, USA) was *p* < 0.05.

## 3. Results and discussion

### 3.1. Mineralization of sea turtle rhamphothecae

Hard alpha-keratin structures have been traditionally described as non-mineralized tissue. Yet, calcium phosphate has been previously detected in whale baleen and white rhinoceros horns [19,20,38,40] and has been linked to maintaining structural integrity and increasing the capacity to withstand mechanical stresses. Similarly, mineral has been predicted to reinforce bird and turtle rhamphotheca β-keratin, although to our knowledge no work has been conducted to quantify mineral densities of these feeding apparatuses [21,41]. To determine the presence and density of mineral in sea turtle rhamphothecae, we calculated BMD from the portion of the upper and lower keratinous beaks that are involved in the processing of prey (figure 2*b,f*). Although animal sizes for each species varied by several centimeters (SCL for loggerheads: 59.5–84.4 cm and Kemp's ridleys: 28.5–62.5), simple regressions showed that SCL was not a significant predictor for BMD.

**Figure 4.**
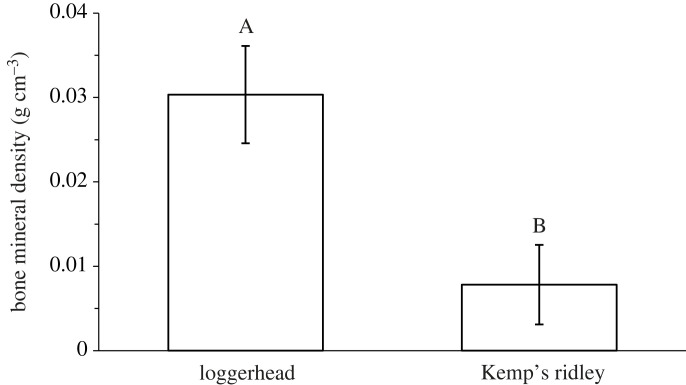
BMD (g cm^−3^) measurements from the upper and lower rhamphothecae of each study species. Loggerheads had a significantly greater BMD than Kemp's ridleys. Error bars denote s.e.

Supporting our hypothesis, loggerheads (*x̄* = 0.03 ± 0.006) had significantly greater BMD throughout the keratinous matrix of their rhamphothecae than Kemp's ridley (*x̄* = 0.008 ± 0.005) sea turtles (*U* = 2.53, *p* = 0.0113; figure 4). Interestingly, while all loggerhead specimens were mineralized, only three out of the eight Kemp's ridleys had detectable mineralization (table 1). Mineralization varied between the upper and lower rhamphothecae for every individual. Of the three Kemp's ridleys with BMD values, one animal only had mineral in its lower rhamphotheca (STR20210509-001), and loggerheads 20210424VRG-01 and LNH110719-01 only had mineral in their upper and lower rhamphothecae, respectively (table 1).

**Table 1.** Rhamphothecae bone mineral density among individual loggerhead and Kemp's ridley sea turtles. UR = upper rhamphotheca; LR = lower rhamphotheca; SCL = standard carapace length, BMD = bone mineral density.

species	animal number	SCL (cm)	UR BMD (g cm^−3^)	LR BMD (g cm^−3^)
loggerhead	1	84.4	0.019	0.018
	2	78	0.069	0.059
	3	78	0.064	0
	4	72.3	0.04	0.034
	5	61.4	0	0.029
	6	60.2	0.031	0.032
	7	59.5	0.018	0.014
Kemp's ridley	1	62.5	0	0
	2	61.9	0.026	0.073
	3	58.8	0	0
	4	49.2	0	0
	5	42.6	0	0
	6	33.2	0	0
	7	29.5	0	0.01
	8	28.5	0.013	0.003

Interspecific differences in calcification levels have been previously found in hard keratins of other taxa. Szewciw *et al*. [20] found that a sei whale's (*Balaenoptera borealis*) baleen had approximately 5*×* greater calcium and phosphorus levels than a humpback whale (*Megaptera novaeangliae*), which had 2.5*×* more of each mineral compared with minke whales (*Balaenoptera acutorostrata*). Since sei whales filter feed on some of the smallest prey among rorquals, a high density of calcium phosphate in baleen may support bristle separation and subsequently decrease filtering porosity [20]. Like whale baleen, sea turtle rhamphothecae have little or no opportunity to fully dry throughout their lifetimes, and the presence of calcium phosphates throughout the keratinous matrix may aid in stiffening the structure. However, considering the present study's findings, one may conclude that rhamphotheca mineralization is not essential since several Kemp's ridley specimens had no calcium phosphate as measured by BMD here (table 1 and figure 4).

### 3.2. Rhamphotheca water content among varying conditions

**Figure 5.**
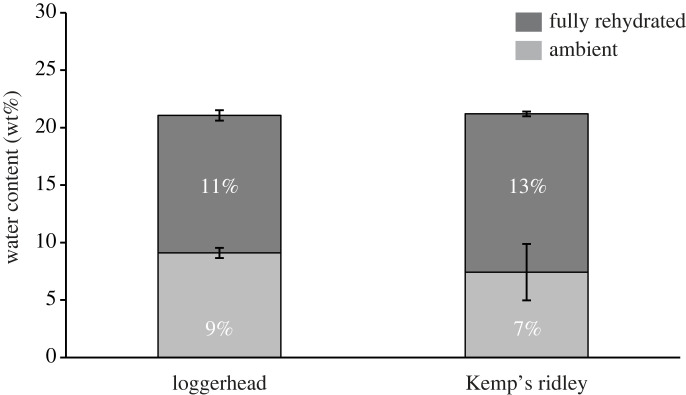
Water content (wt %) of rhamphothecae in the ambient dry and fully hydrated conditions.

Hydration levels of hard keratin correlate strongly with material behaviour, and the capacity of hard keratin for total water absorption can vary among taxa [23,24,42]. In the present study, rhamphothecae from three loggerhead and three Kemp's ridley sea turtles were assessed to determine water content for each species. Total water content, as well as water weight between fully dehydrated and ambient dry conditions, and between ambient dry and fully hydrated states, was calculated. No significant difference among loggerhead and Kemp's ridley rhamphotheca dehydration capacity, hydration capacity and total water content were found (figure 5; *F* = 1.69, *p* = 0.2260). Since two salvaged loggerhead specimens had missing morphometric data, we were unable to run species-specific simple regressions between SCL and water content data (table 2).

**Table 2.** Rhamphothecae water content in fully dehydrated, ambient dry and fully rehydrated conditions. U/L = upper/lower rhamphotheca; SCL = standard carapace length; FDW = fully dehydrated weight; ADW = ambient dry weight; FHW = fully hydrated weight; ** = estimated SCL based on other morphological measurements.

species	animal number	SCL (cm)	U/L	FDW (g)	ADW (g)	FHW (g)
loggerhead	5	61.4	U	14.4	16	17.7
			L	10	11.1	12.3
	8	> 60**	U	57.8	62.8	69.1
			L	36.2	39.9	44.2
	9	> 60**	U	13.6	14.9	17.7
			L	11.1	12.2	14.9
Kemp's ridley	1	62.5	U	13.3	14.2	16.5
			L	9.3	10.2	11.8
	3	58.8	U	12.1	12.9	14.9
			L	9	9.7	11.2
	6	33.2	U	4.5	4.9	5.7
			L	3.3	3.6	4.2

Apart from nesting and basking behaviours, sea turtles are almost consistently submerged in water [43]. Thus, it is reasonable to assume their rhamphotheca keratin may have a greater capacity to hold water than the hard keratins of terrestrial animals. Our results supported the contrary: the 20% water content of sea turtle rhamphotheca keratin was less than half that of pronghorn (*Antilocapra americana*; 52%) and bighorn sheep (*Ovis canadensis*; 40%) horn keratin [23]. Perhaps, it is *because of* sea turtles' constant submergence in water that keratin water absorption capacity must be limited. If the fully hydrated water content were at 40–52% as in terrestrial bovid horn, material stiffness and structural integrity could be compromised, since the constant state of sea turtle rhamphothecae is fully hydrated. Conversely, bovid horn keratin, which is mostly exposed to ambient dry conditions, does not operate at full hydration, but probably at hydration levels slightly above ambient dry due to the physiology of the underlying bone [41,44]. If too dry, bovid horn keratin can be hyper-susceptible to fracture [41]. These varying hydration levels could potentially be mitigated by keratin's microarchitecture, such as tubular density (porosity), which has been positively correlated with hydration levels in bovid horn [23]. If keratin tubules and nanopores are serving a similar function as water reservoirs in sea turtle rhamphothecae, the present data could be used to inform future investigations employing the use of high magnification optical micrographs to quantify porosity.

Water absorption capabilities of sea turtle rhamphothecae differed from those of keratinous structures from marine mammals, specifically that of whale baleen. When Werth *et al*. [24] assessed the increase in whale baleen between ambient dry and fully hydrated conditions, they found that North Atlantic right whales (*Eubalaena glacialis*) and bowhead whales (*Balaena mysticetus*) had an average of 32% and 34% water content, respectively. These values were drastically less for sea turtles; between ambient dry and fully hydrated conditions, loggerheads had only 11% water content while Kemp's ridleys had 13% (table 2 and figure 5). Although both baleen and rhamphothecae are used for aquatic foraging, their associated mechanisms of feeding, and the resulting forces they are subjected to, differ greatly. Whale baleen are long structures with high surface area that must be stiff enough to resist drag forces, yet flexible enough to behave as a dynamic filter by governing intra-oral flow fields [24,45]. Conversely, sea turtle rhamphothecae are relatively compact and streamlined structures that have fewer selective pressures to behave pliantly. Sea turtles use beaks to shear through grasses and algae; to acquire, manipulate, cut and swallow soft-bodied prey whole; and to crush hard prey items [10,46,47]. Thus, the low water content of fully hydrated rhamphothecae may reflect the requirement for a stiffer structure for these feeding behaviours [12,48,49].

### 3.3. Compression tests

Since hydration level influences keratin material behaviour and full hydration is the biologically relevant condition for sea turtle rhamphotheca keratin, we immersed samples (*n* = 39) from six loggerheads (SCLs = 59.5–84.4; *x̄* = 72.1 ± 4.17) and four Kemp's ridleys (SCLs = 42.6–61.9; *x̄* = 52.4 ± 4.16) in filtered seawater for 5 days before compression testing [20,23]. Contrary to our hypothesis, we found no significant differences among loggerhead and Kemp's ridley keratin's Young's modulus, yield strength or toughness (table 3; *F* = 0.13, *p* = 0.6413). Since species-specific simple regressions between SCL and material properties were not significant, we confirmed that animal size was not a predictor for compressive mechanical properties in this study.

**Table 3.** Compressive mechanical properties of sea turtle rhamphothecae.

	species	
	loggerhead	Kemp's ridley
Young's modulus (MPa)	902.41 ± 130.835	1065.2 ± 151.259
yield strength (MPa)	31.92 ± 5.538	26.42 ± 6.041
toughness 50% (MJ m^−3^)	24.24 ± 2.615	20.30 ± 1.707

Loggerhead sea turtles are capable of feeding on especially hard prey items using bite forces reaching up to 1766 N; we predicted that the Young's modulus, yield strength and toughness of their rhamphotheca keratin would surpass those of Kemp's ridleys. Due to the association between material behaviour and the presence of mineral, this hypothesis was reinforced by our finding that loggerhead rhamphothecae had not only an overall greater mineral density, but a higher frequency of mineral presence among individuals (table 1). Our results suggest that, while calcium phosphate may contribute to mechanical properties, this relationship is not straightforward, since other mechanisms may also be at work. One potential mechanism could be an increase in sulfur concentration [20,50]. Previous work has shown that sulfur-rich keratins with a high amount of sulfur cross-links increased material rigidity and durability [18,51–53]. When Szewciw *et al*. [20] analysed whale baleen material composition with atomic absorption spectroscopy (AAS), they found interspecific trade-offs between concentrations of calcium phosphate and sulfur: although the Young's modulus and yield strength of sei whale and humpback whale baleen were comparable, sei whales had much higher calcium phosphate than sulfur, while the opposite trend was found in humpbacks. Future studies on sea turtle rhamphothecae should employ AAS to investigate if there are similar trade-offs between calcium phosphate and sulfur concentrations.

Loggerhead and Kemp's ridley sea turtle bite forces must sufficiently crush through the hard chitin and calcium carbonate-rich exoskeletons of their prey without incurring damage to their feeding apparatus [6,7]. Blue crabs (*Callinectes sapidus*), a common prey item for both loggerheads and Kemp's ridleys, have carapaces which require forces between 30 and 490 N to be crushed [54]. Similarly, venus clams (*Callista chione*), which are predated upon by loggerheads, are broken with an average load of 300 N [55,56]. These values are within the lower range of bite forces from neritic zone-inhabiting, durophagous loggerheads (greater than 60 cm SCL) [10]. Load data from the present study suggest that loggerhead rhamphotheca keratin is not damaged due to their high bite forces; during compression tests, keratin did not plastically (i.e. permanently) deform until 850–1550 N (1288 ± N). Although we lack bite force data for Kemp's ridleys, we found that their rhamphotheca keratin didn't deform until compressed to 500–1400 N (858 ± N), suggesting that their bite forces are high enough to support their durophagous predation upon most crabs without physically compromising their rhamphothecae.

Interestingly, the forces required to damage rhamphotheca keratin are similar to those that fracture teeth; Sheen *et al*. [57] found that human molars fracture at average loads of only 1000 N. However, while few tetrapods can replace worn-down or broken teeth, the cornified rhamphotheca keratin of birds and turtles grows and wears continuously throughout life [58]. These comparisons among toothed and beaked feeding apparatuses can provide insight into the evolutionary advantages of keratinous beaks for durophagous diets.

To consider the compressive mechanical properties of rhamphothecae among beaked taxa, we compared Young's modulus data from the present study (table 3) with previously published data on the rhamphothecae from two bird species. We found that the values were consistent for: loggerheads (0.9 GPa), toco toucans (*Ramphastos toco*; 1 GPa), Kemp's ridleys (1.1 GPa), and wreathed hornbills (*Rhyticeros undulatus*; 1.2 GPa) [7]. While the sea turtle samples were mechanically tested in a fully hydrated condition, Seki *et al*. [7] tested their hornbill and toucan keratin in an ambient dry state, which usually results in greater material stiffness [23]. Together, these data suggest that cornified beak keratin is specifically adapted for either a terrestrial or aquatic environment. Factors such as hydration level mitigation may vary between environments in a way that results in similar material behaviours of keratinous feeding apparatuses.

Feeding apparatuses are structures whose performance is informed not only by material properties, but also by shape and size [59]. Previous studies have linked beak morphology to feeding ecology; Soons *et al*. [60] and Olsen [61] found that beak shapes have evolved to resist feeding forces when processing hard items in finches and to support increasing levels of herbivory in waterfowl, respectively. Therefore, we propose that future investigations use geometric morphometric and finite-element analyses to understand the extent in which rhamphothecal morphology determines the diverse feeding performances of species in these edentulous, aquatic taxa.

## 4. Conclusion

Since feeding is directly connected to fitness, the considerable radiation and evolutionary success of extinct and extant birds and Testudines (i.e. turtles, tortoises and terrapins) is likely in part due to the innovation of replacing teeth with a cornified beak [62]. Despite the considerably lower stiffness of rhamphotheca keratin compared with tooth enamel, various species of turtles are durophagous. Here, we present evidence that sea turtle rhamphothecae are mineralized, and this is especially noticeable in the loggerhead species. In support of our hypothesis, we found that loggerhead turtles, who have greater theoretical bite forces (informed by head morphometrics and muscle size), had greater BMD compared with the Kemp's ridley turtles. Despite having different BMDs, the rhamphotheca compressive mechanical properties of both species were statistically similar. In this study we did not find a relationship between keratin mineralization and mechanical properties; however, it is possible that mineralization, in conjunction with other material characteristics (e.g. an increase in sulfur concentration), influence overall material behaviour. Material composition, in conjunction with a low capacity for water absorption, may be tuned to provide rhamphothecae with the necessary properties to endure the mechanical stresses of durophagy.

## Data Availability

All data presented here are accessible in the Open Science Framework repository (doi:10.17605/OSF.IO/84RXG) [63]. The data are provided in the electronic supplementary material [64].
